# Targeted inhibition of mitochondrial Hsp90 suppresses localised and metastatic prostate cancer growth in a genetic mouse model of disease

**DOI:** 10.1038/bjc.2011.9

**Published:** 2011-02-01

**Authors:** B H Kang, M Tavecchio, H L Goel, C-C Hsieh, D S Garlick, C M Raskett, J B Lian, G S Stein, L R Languino, D C Altieri

**Affiliations:** 1 Prostate Cancer Discovery and Development Program, 3601 Spruce Street, Philadelphia, PA 19104, USA; 2 Wistar Institute Cancer Center, 3601 Spruce Street, Philadelphia, PA 19104, USA; 3 Department of Cancer Biology, University of Massachusetts Medical School, Worcester, MA 01605, USA; 4 Department of Cell Biology, University of Massachusetts Medical School, Worcester, MA 01605, USA; 5 Department of Cancer Biology, Kimmel Cancer Center, Thomas Jefferson University, Philadelphia, PA 19107, USA

**Keywords:** prostate cancer, metastasis, mitochondria, Hsp90, TRAMP

## Abstract

**Background:**

The molecular chaperone heat shock protein-90 (Hsp90) is a promising cancer drug target, but current Hsp90-based therapy has so far shown limited activity in the clinic.

**Methods:**

We tested the efficacy of a novel mitochondrial-targeted, small-molecule Hsp90 inhibitor, Gamitrinib (GA mitochondrial matrix inhibitor), in the Transgenic Adenocarcinoma of the Mouse Prostate (TRAMP) model. The TRAMP mice receiving 3-week or 5-week systemic treatment with Gamitrinib were evaluated for localised or metastatic prostate cancer, prostatic intraepithelial neoplasia (PIN) or localised inflammation using magnetic resonance imaging, histology and immunohistochemistry. Treatment safety was assessed histologically in organs collected at the end of treatment. The effect of Gamitrinib on mitochondrial dysfunction was studied in RM1 cells isolated from TRAMP tumours.

**Results:**

Systemic administration of Gamitrinib to TRAMP mice inhibited the formation of localised prostate tumours of neuroendocrine or adenocarcinoma origin, as well as metastatic prostate cancer to abdominal lymph nodes and liver. The Gamitrinib treatment had no effect on PIN or prostatic inflammation, and caused no significant animal weight loss or organ toxicity. Mechanistically, Gamitrinib triggered acute mitochondrial dysfunction in RM1 cells, with loss of organelle inner membrane potential and release of cytochrome-*c* in the cytosol.

**Conclusions:**

The Gamitrinib has pre-clinical activity and favourable tolerability in a genetic model of localised and metastatic prostate cancer in immunocompetent mice. Selective targeting of mitochondrial Hsp90 could provide novel molecular therapy for patients with advanced prostate cancer.

Despite advances in treating early-phase prostate cancer (Carter *et al*, 2006), advanced disease, characterised by castration resistance and bone metastases, poses significant therapeutic challenges (Taichman *et al*, 2007), with over 30 000 deaths in the United States alone. Cytotoxic or radiation has limited efficacy in these patients, and molecular therapies are still in early stages of evaluation (Vogiatzi *et al*, 2009). Because advanced prostate cancer is heterogeneous (Taylor *et al*, 2010), targeting so-called ‘nodal’ cancer genes (Lamb *et al*, 2006) overseeing multiple downstream pathways of tumour maintenance (Butcher, 2005) may offer concrete therapeutic prospects. In this context, the molecular chaperone heat shock protein-90 (Hsp90) is a nodal cancer gene (Whitesell and Lindquist, 2005), controlling the folding and/or maturation of client proteins involved in tumour cell proliferation, survival and adaptation (Trepel *et al*, 2010). The Hsp90 has been intensely pursued for cancer therapeutics, and several small-molecule antagonists of its ATPase pocket have been developed (Drysdale and Brough, 2008). However, Hsp90-based therapy (Kim *et al*, 2009) has shown so far modest activity in patients with epithelial malignancies, including prostate cancer (Heath *et al*, 2008), whether as single agent (Solit *et al*, 2008) or combined with cytotoxics (Tse *et al*, 2008) or molecular therapies (Modi *et al*, 2007).

A key feature of Hsp90 and Hsp90-like molecules is their localisation to multiple subcellular compartments (Trepel *et al*, 2010). Recently, a pool of Hsp90 has been found in mitochondria of tumour cells (Kang *et al*, 2007), where it promotes cell survival by antagonising cyclophilin D (CypD)-dependent organelle permeability transition and apoptosis (Green and Kroemer, 2004). Whether this pathway (Kang *et al*, 2007) influences the response to Hsp90-based therapy in the clinic (Trepel *et al*, 2010) is currently unknown, but it is intriguing that none of the Hsp90 ATPase antagonists currently in (pre)clinical development (Drysdale and Brough, 2008) has the ability to accumulate in mitochondria (Kang *et al*, 2009), thus leaving unscathed this general survival mechanism. To address this limitation, a new class of small-molecule Hsp90 inhibitors selectively targeted to mitochondria, that is, Gamitrinibs (GA mitochondrial matrix inhibitors) was recently generated (Kang *et al*, 2009), which exhibited potent anti-cancer activity in various xenograft tumour models, *in vivo* (Kang *et al*, 2009).

In this study, we evaluated the pre-clinical activity of Gamitrinib in the Transgenic Adenocarcinoma of the Mouse Prostate (TRAMP) model (Greenberg *et al*, 1995). Albeit with limitations (Pienta *et al*, 2008), prostatic tumourigenesis in TRAMP mice recapitulates many aspects of the human disease on an immunocompetent background (Kaplan-Lefko *et al*, 2003), and is suitable for cancer drug discovery, *in vivo* (Zorn *et al*, 2007).

## Materials and Methods

### Cells and reagents

RM1 cells derived from TRAMP mice have been described (Voeks *et al*, 2002). The chemical synthesis, HPLC profile and mass spectrometry of mitochondria-targeted small-molecule Hsp90 antagonists, Gamitrinibs, have been reported (Kang *et al*, 2009). This study utilised Gamitrinib–G4 (G–G4), which contains the Hsp90 ATPase inhibitory structure of 17-AAG (LC-Laboratories, Woburn, MA, USA) linked to four tandem repeats of guanidinium, used as a mitochondriotropic moiety (Kang *et al*, 2009).

### The Gamitrinib treatment of TRAMP mice

All experiments involving animals were approved by an Institutional Animal Care and Use Committee. The TRAMP model has been described (Kaplan-Lefko *et al*, 2003), and involves expression of the SV40 large T and small t oncogene in the prostatic epithelium under the control of the minimal −426/+28 rat probasin promoter (Greenberg *et al*, 1995). Transgene expression is regulated by androgen, and results in a spectrum of lesions, including prostatic intraepithelial neoplasia (PIN), invasive adenocarcinoma, neuroendocrine tumours and metastases to loco-regional abdominal lymph nodes, liver and lungs (Greenberg *et al*, 1995; Kaplan-Lefko *et al*, 2003). Female TRAMP mice on a C57BL/6 background were bred with non-transgenic males, and the offspring was weaned at 3–4 weeks of age. Male pups were genotyped by PCR amplification of tail genomic DNA using transgene-specific primers. To test a potential anti-cancer activity of Gamitrinib (Kang *et al*, 2009) in this model, TRAMP mice were divided into two age groups to receive G–G4 monotherapy at 5 mg/kg in cremophor as i.p. injections, with the schedule 3 days on/2 days off. Mice in group 1 (short-term treatment) received G–G4 starting at 21.9 weeks of age for 3 weeks (24.9 weeks of age), with analysis of primary and metastatic prostate cancer as end point. Animals in group 2 (long-term treatment) were started on G–G4 at 14.7 weeks of age for 5 weeks (19.7 weeks of age) and assessed histologically for primary prostate cancer growth, PIN and localised inflammation.

### Magnetic resonance imaging

The 2T magnetic resonance imaging (MRI) analysis of TRAMP mice was carried out on a Bruker/General Electric CSI-II 2.0 T/45 cm imaging spectrometer (Madison, WI, USA) equipped with a thermostat-controlled animal holder and gas anaesthesia, containing magnetic field gradients, RF phase, amplitude control and automatic shimming. For these experiments, 20-week-old non-castrated TRAMP mice were imaged by MRI using the following parameters: repetition time (TR)=2000/600 ms; echo time (TE)=25 ms; data acquisition field-of-view=40 mm × 40 mm/30 mm × 30 mm; slice thickness (ST)=1 mm; data acquisition matrix =256 × 256; number of echo averages=4; and display FO=30 mm × 30 mm.

### Analysis of mitochondrial dysfunction

RM1 cells isolated from TRAMP prostate tumours (Voeks *et al*, 2002) were incubated with 20 *μ*M G–G4 or non-mitochondrially targeted Hsp90 inhibitor, 17-AAG, and analysed after 12 h for changes in mitochondrial membrane potential by JC-1 (200 *μ*M) staining and multiparametric flow cytometry on a FACSCalibur (Becton Dickinson, Franklin Lake, NJ, USA), as described (Kang *et al*, 2009). Alternatively, cytosolic extracts were isolated from treated RM1 cells using an ApoAlert Cell Fractionation Kit (Clontech, Otsu, Shiga, Japan), and analysed by western blotting.

### Histology

The TRAMP mice in control or G–G4-treated groups were killed, and the entire genitourinary tract containing seminal vesicles, prostate (including dorsal, lateral, ventral and anterior lobes) and urethra (thus excluding the urinary bladder), was isolated, fixed and stained with hematoxylin/eosin. In some experiments, tissue sections were stained with an antibody to the proliferation-associated marker, Ki67, as described (Kang *et al*, 2009). At the end of the experiment, organs from control or G–G4-treated TRAMP mice were removed, paraffin embedded and analysed by H&E staining and light microscopy. The histological analysis in each case was performed by a veterinary pathologist (DSG), and the percentage of prostate gland involvement with PIN, adenocarcinoma or neuroendocrine tumours was assessed in individual prostatic lobes. The scoring system used to quantify inflammation or metastatic prostate cancer was as follows: 0, none; 1, mild; 2, moderate; and 3, marked.

### Statistical analysis

Data were analysed using the unpaired *t*-test on a GraphPad software package (Prism 4.0, La Jolla, CA, USA) for Windows. All statistical tests were two sided. A *P*-value of 0.05 was considered to be statistically significant.

## Results

### Prostate tumourigenesis in TRAMP mice

We began this study by quantifying prostate cancer growth in untreated TRAMP mice (Greenberg *et al*, 1995). Consistent with previous reports (Kaplan-Lefko *et al*, 2003), TRAMP mice at 22 weeks of age exhibited enlarged prostates, by MRI (Supplementary Figure 1). Prostatic lesions under these conditions included well-differentiated adenocarcinomas with low proliferative index as well as large neuroendocrine tumours, composed of sheets of small, undifferentiated cells that stained intensely for the proliferation-associated marker, Ki-67 (Supplementary Figure 1).

### Gamitrinib inhibits localised prostate cancer growth in TRAMP mice

**Figure 1:**
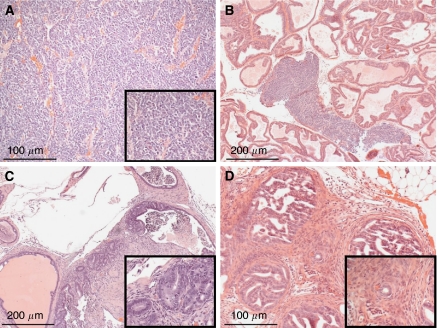
Prostate histopathology of untreated TRAMP mice at 19.7 weeks of age (group 2). Prostatic samples were isolated from group 2 TRAMP mice, and analysed by H&E staining and light microscopy. Representative cases of prostatic neuroendocrine tumours (**A**, **B**; ventral prostate) or adenocarcinoma (**C**, **D**; dorsal prostate) in group 2 TRAMP mice associated with extensive PIN lesions and various degrees of inflammation are shown.

Consistent with these data, prostatic samples from untreated TRAMP mice harvested at 19.7 weeks of age (group 2) revealed the presence of neuroendocrine tumours, adenocarcinomas and PIN lesions, mixed with various degrees of local inflammation (Figure 1). Large neuroendocrine tumours occupying >50% of a prostatic lobe were observed in dorso-lateral and ventral prostate samples (Figure 1A and B), whereas adenocarcinomas (Figure 1C and D) were histologically well differentiated, of smaller size, that is, 5–25% of a prostatic lobe and equally distributed in dorso-lateral, ventral and anterior prostate. A complete histopathological analysis of control group 2 TRAMP mice is presented in Table 1.

**Figure 2:**
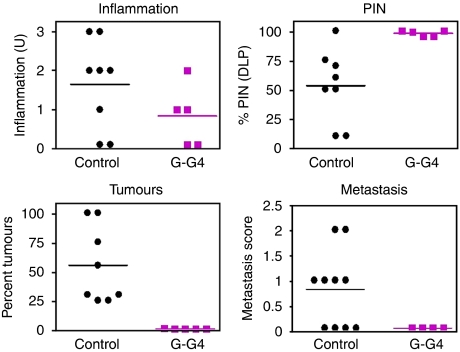
Quantification of prostatic lesions in untreated or Gamitrinib-treated TRAMP mice. Prostatic samples were harvested from untreated (control) or Gamitrinib (G-G4)-treated TRAMP mice, and analysed by H&E staining and light microscopy. The percentage of PIN lesions in representative matched samples of dorso-lateral prostate (DLP) from the various groups is shown. An inflammation or metastasis score was determined, and expressed as arbitrary units (U). Quantification of inflammation (NS), PIN (*P*=0.091) or tumour formation (*P*=0.0038) was carried out in TRAMP mice at 19.7 weeks of age (group 2). Quantification of metastasis to liver and loco-regional abdominal lymph nodes was determined in TRAMP mice at 24.9 weeks of age (group 1). Abbreviation: NS=not significant.

Long-term treatment of group 2 TRAMP mice with G–G4 suppressed the growth of localised prostate cancer of both neuroendocrine and adenocarcinoma origin (Supplementary Figure 2 and Figure 2). Conversely, G–G4 treatment had no effect on localised prostatic inflammation in TRAMP mice, whereas it moderately but significantly increased the incidence and distribution of PIN lesions compared with age-matched control TRAMP mice (Supplementary Figure 2 and Figure 2). A complete histopathological characterisation of Gamitrinib-treated group 2 TRAMP mice is shown in Table 2.

### Gamitrinib inhibits metastatic prostate cancer in TRAMP mice

Histological analysis of untreated group 1 TRAMP mice at 24.9 weeks of age revealed the presence of large neuroendocrine tumours in the dorso-lateral and ventral prostate, and well-differentiated adenocarcinomas in various prostatic lobes (Table 3). In all, 6 out of 10 of these animals also presented moderate prostate cancer dissemination to liver and loco-regional abdominal lymph nodes (Table 3 and Figure 2), consistent with previous observations (Hsieh *et al*, 2007). In contrast, none of the age-matched G–G4-treated animals in group 1 (0 out of 4 mice) had metastatic prostate cancer in liver or abdominal lymph nodes (Figure 2). Histological examination of brain, kidneys or lungs in these mice was also negative (not shown).

### Safety of long-term Gamitrinib treatment in TRAMP mice

Both groups of TRAMP mice given Gamitrinib exhibited no significant weight loss throughout treatment (Supplementary Figure 3A). Similarly, organs harvested at the end of treatment from group 2 TRAMP mice were histologically unremarkable (Supplementary Figure 3B) compared with age-matched untreated mice (not shown).

### ‘Mitochondriotoxic’ activity of Gamitrinib

**Figure 3:**
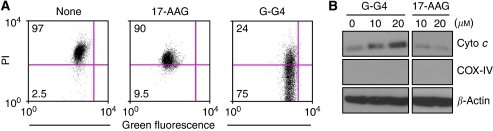
‘Mitochondriotoxic’ activity of Gamitrinib. (**A**) The TRAMP tumour-derived RM1 cells were labelled with the mitochondrial membrane potential-sensitive dye, JC1, incubated as indicated and analysed after 12 h by multiparametric flow cytometry. The percentage of cells in each quadrant is indicated. (**B**) RM1 cells were treated as indicated, and isolated cytosolic extracts were analysed by western blotting. COX-IV and *β*-actin were used as mitochondrial or cytosolic markers, respectively. Abbreviations: Cyto-*c*=cytochrome-*c*; PI=propidium iodide.

To begin elucidating the mechanism of anti-cancer activity of Gamitrinib in the TRAMP model, we next used RM1 cells that are derived from TRAMP tumours (Voeks *et al*, 2002). Treatment of RM1 cells with Gamitrinib caused nearly complete loss of mitochondrial inner membrane potential, as detected by multiparametric flow cytometry (Figure 3A). This was associated with concentration-dependent release of mitochondrial cytochrome-*c* in the cytosol of Gamitrinib-treated RM1 cells (Figure 3B). Conversely, non-subcellularly targeted 17-AAG had no effect on mitochondrial membrane potential or cytochrome-*c* release (Figures 3A and B).

## Discussion

In this study, we have shown that systemic administration of Gamitrinib (Kang *et al*, 2009), a novel small molecule that targets exclusively the pool of Hsp90 in mitochondria (Kang *et al*, 2007), suppressed localised and metastatic prostate cancer growth in TRAMP mice (Greenberg *et al*, 1995), with no effect on PIN or local inflammation. Long-term systemic treatment of TRAMP mice with Gamitrinib was feasible, with no evidence of systemic or organ toxicity. Mechanistically, Gamitrinib functioned as a ‘mitochondriotoxic’ agent in the TRAMP model, triggering loss of organelle inner membrane potential and discharge of cytochrome-*c* in the cytosol.

Although still the backbone of cancer drug discovery, xenograft studies in immunocompromised mice have significant drawbacks (Kelland, 2004), as tumour growth in these settings does not recapitulate the complexity of clonal selection, cross-talk with the microenvironment, interplay of inflammatory responses and acquisition of metastatic traits. This has prompted renewed interest in exploiting genetically engineered mouse models for cancer drug discovery (Walrath *et al*, 2010), especially for prostate cancer, where cross-talk between the tumour cell population and its microenvironment has a critical role in progression to castration resistance and metastasis (Taichman *et al*, 2007). Despite its well-known limitations (Pienta *et al*, 2008), including the preponderance of neuroendocrine tumours compared with adenocarcinoma (Chiaverotti *et al*, 2008), and the failure to metastasise to bones (Hsieh *et al*, 2007), prostatic tumourigenesis in TRAMP mice (Shappell *et al*, 2004) has provided a reliable genetic model for the human disease (Kaplan-Lefko *et al*, 2003), suitable for pre-clinical studies (Zorn *et al*, 2007).

Here, the anti-cancer activity of Gamitrinib in TRAMP mice extends recent studies in xenograft models (Kang *et al*, 2009), including prostate cancer, where systemic administration of Gamitrinib-TPP (Kang *et al*, 2009) suppressed the growth of subcutaneous or bone-localised PC3 prostate tumours in immunocompromised mice (Kang *et al*, 2010). In the TRAMP model, Gamitrinib-G4, which contains a structurally distinct mitochondrial-targeting moiety compared with Gamitrinib-TPP (Kang *et al*, 2009), was active across the spectrum of poorly differentiated, rapidly proliferating neuroendocrine tumours, as well as of differentiated adenocarcinoma. This is consistent with the abundant distribution of one of the targets of Gamitrinibs (Kang *et al*, 2007), the mitochondrial Hsp90 homologue TNF receptor-associated protein-1 (Trepel *et al*, 2010), in all Gleason grade localised and metastatic prostate cancer in humans, but not benign prostatic hyperplasia (Leav *et al*, 2010).

Consistent with earlier observations (Kang *et al*, 2009), the mechanism of action of Gamitrinib in the TRAMP model involved acute induction of mitochondrial dysfunction (Kang *et al*, 2010), with loss of organelle inner membrane potential and release of cytochrome-*c* in the cytosol (Green and Kroemer, 2004). This produces direct tumour cell killing by Gamitrinib, at variance with the mainly cytostatic activity of non-subcellularly targeted Hsp90 inhibitors (Kang *et al*, 2009). In prostate cancer, Gamitrinib-mediated killing indistinguishably affected androgen-dependent and -independent cell types (Kang *et al*, 2010; Leav *et al*, 2010), which may contribute to its activity against TRAMP tumours, often characterised by loss of androgen receptor (Huss *et al*, 2007) and androgen insensitivity (Kaplan-Lefko *et al*, 2003). With respect to the anti-metastatic activity of Gamitrinib in the TRAMP model, it is possible that prostate cancer cells in the hypoxic environment of a metastatic niche, enriched in reactive oxygen species (Sung *et al*, 2008), may become especially ‘addicted’ to cytoprotection by mitochondrial Hsp90s (Kang *et al*, 2007). This model is consistent with an important role of CypD (Baines *et al*, 2005; Nakagawa *et al*, 2005) in mediating oxidative stress-induced mitochondrial permeability transition (Hua *et al*, 2007; Montesano Gesualdi *et al*, 2007), a cell death response antagonised by mitochondrial Hsp90s (Kang *et al*, 2007).

Long-term, continuous Gamitrinib treatment of TRAMP mice was feasible, devoid of systemic or organ side effects, *in vivo*. This tolerability likely reflects the low to undetectable expression of the targets of Gamitrinib, that is, mitochondrial Hsp90s, in most normal tissues, as opposed to tumours (Kang *et al*, 2007). This cytoprotective pathway may be also uniquely ‘wired’ in tumour cells, as suggested by the insensitivity of normal prostatic epithelium to Gamitrinib-mediated killing (Leav *et al*, 2010) and the lack of association between Hsp90s and CypD in mitochondria of normal tissues (Ghosh *et al*, 2010).

In sum, we have shown that one of the Gamitrinib variants, G–G4 (Kang *et al*, 2009), has activity in a pre-clinical genetic model of localised and metastatic prostate cancer in an immunocompetent background (Greenberg *et al*, 1995). Although additional work is required to define the drug-like properties of Gamitrinibs in anticipation of human testing, the data presented here suggest that selective suppression of mitochondrial Hsp90s may provide novel molecular therapy in prostate cancer, and improve the currently limited activity of Hsp90-based therapy in these patients (Trepel *et al*, 2010).

## Figures and Tables

**Table 1:** Prostate histopathology in untreated TRAMP mice age matched to group 2 (19.7 weeks)

**Mouse no.**	**Neuroendocrine (%)**	**Adenocarcinoma (%)**	**PIN (%)**	**Inflammation (Score)**
1137	100 (DLP, VP)	0	10 (AP)	0
1145	100 (DLP, VP)	0	10 (AP)	0
1150	75 (DLP)	0	100 (DLP); 75 (VP); 10 (AP)	+1 (DLP); +2 (VP)
1158	0	25 (DLP)	50 (DLP); 50 (VP); 10 (AP)	+1 (DLP); +1 (VP)
1208	0	25 (DLP); 5 (VP)	50 (DLP); 75 (VP); 10 (AP)	+2 (DLP); +1 (VP)
1211	0	20 (DLP); 5 (AP)	70 (DLP); 100 (VP); 10 (AP)	+1 (VP); +1 (AP)
1219	50 (VP)	5 (DLP)	75 (DLP); 50 (VP); 25 (AP)	+1 (DLP); +1 (AP)
1224	0	10 (DLP); 20 (VP)	60 (DLP); 80 (VP); 25 (AP)	+1 (VP)

Abbreviations: AP=anterior prostate; DLP=dorso-lateral prostate; PIN=prostatic intraepithelial neoplasia; TRAMP=Transgenic Adenocarcinoma of the Mouse Prostate; VP=ventral prostate.

**Table 2:** Prostate histopathology of Gamitrinib–G4-treated TRAMP mice (group 2; 19.7 weeks)

**Mouse no.**	**Neuroendocrine (%)**	**Adenocarcinoma (%)**	**PIN (%)**	**Inflammation (Score)**
2999	0	1 (DLP)	99 (DLP); 20 (VP); 10 (AP)	+1 (VP)
4255	0	0	95 (DLP); 95 (VP); 10 (AP)	+2 (VP)
4260	0	0	100 (DLP); 50 (VP)	+1 (VP)
4464	0	0	95 (DLP); 95 (VP); 25 (AP)	0
4473	0	0	100 (DLP); 50 (VP); 10 (AP)	0

Abbreviations: AP=anterior prostate; DLP=dorso-lateral prostate; Gamitrinib=GA mitochondrial matrix inhibitor; PIN=prostatic intraepithelial neoplasia; TRAMP=Transgenic Adenocarcinoma of the Mouse Prostate; VP=ventral prostate.

**Table 3:** Prostate histopathology of untreated TRAMP mice age matched to group 1 (24.9 weeks)

**Mouse**	**Neuroendocrine (%)**	**Adenocarcinoma**	**Metastasis**
1207	0	Well differentiated (DLP)	0
1377	0	Well differentiated (DLP, AP)	+ (Liver)
1271	0	Well differentiated (DLP, AP)	+ (Liver)
1282	100 (DLP, VP)	Well differentiated (AP)	+ (Liver, lymph nodes)
1295	100 (DLP, VP)	0	+ (Lymph nodes)
1299	100	0	+ (Liver, lymph nodes)
1300	0	Well differentiated (DLP)	+ (Liver)
1265	0	Well differentiated (DLP)	0
1381	0	Well differentiated (AP)	0
1281	0	Well differentiated (DLP, AP)	0

Abbreviations: AP=anterior prostate; DLP=dorso-lateral prostate; TRAMP=Transgenic Adenocarcinoma of the Mouse Prostate; VP=ventral prostate.
