# Supplementary material for: Targeted inhibition of mitochondrial Hsp90 suppresses localised and metastatic prostate cancer growth in a genetic mouse model of disease
Source: Br J Cancer. 2011 Feb 1;104(4):629–34. doi: 10.1038/bjc.2011.9 (PMC3049604; doi:10.1038/bjc.2011.9)
Supplement: Supplementary Material [file bjc20119x1.doc]

**Rev. Ms. BJC TH/2010/4295 (421)**

**TARGETED INHIBITION OF MITOCHONDRIAL Hsp90 SUPPRESSES LOCALIZED AND METASTATIC PROSTATE CANCER GROWTH IN A GENETIC MOUSE MODEL OF DISEASE**

Byoung Heon Kang, Michele Tavecchio, Hira Lal Goel, Chung-Cheng Hsieh, David S. Garlick, Christopher M. Raskett, Jane B. Lian, Gary S. Stein, Lucia R. Languino, and Dario C. Altieri

**SUPPLEMENTARY MATERIAL**

**
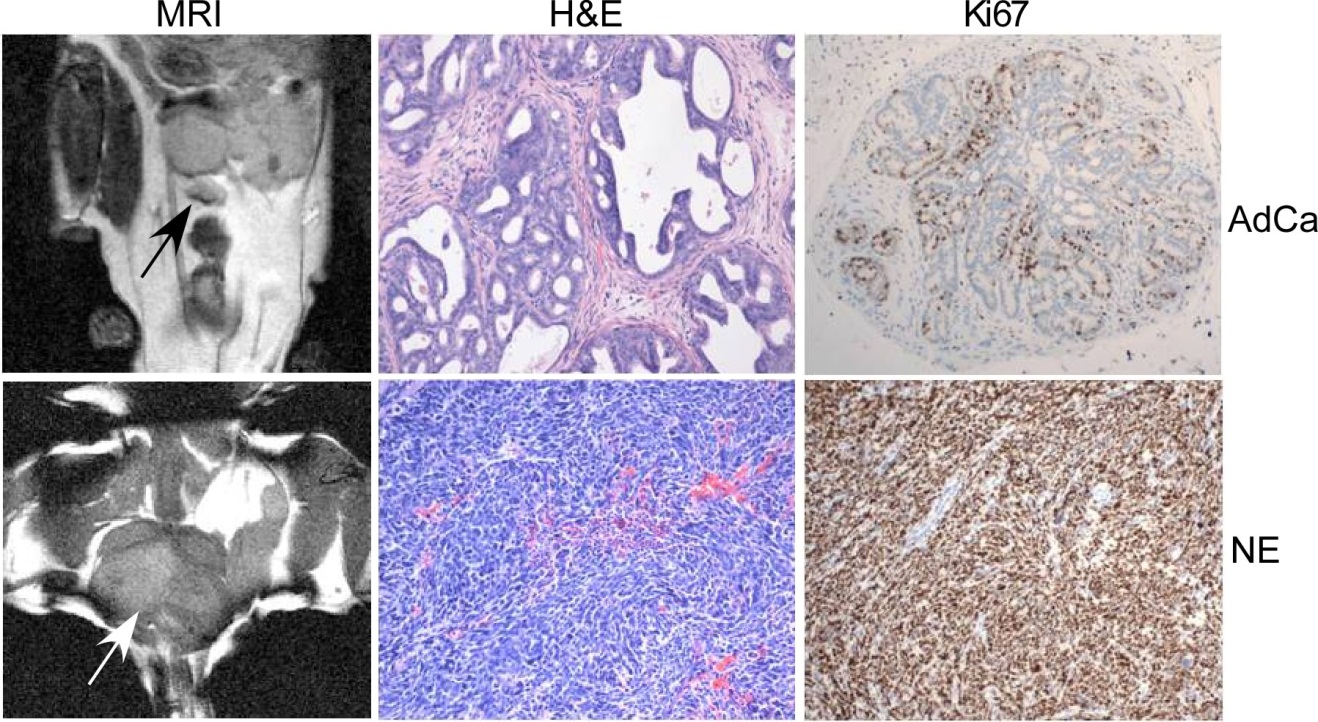
**

**Supplementary Figure 1**. Prostatic tumorigenesis in the TRAMP mouse model. Twenty-two weeks old TRAMP mice were analyzed by MRI (*left*), histology by H&E staining (*middle*) or expression of the proliferation-associated marker, Ki-67 (*right*), by immunohistochemistry. *Arrows*, prostatic tumors. AdCa, adenocarcinoma; NE, neuroendocrine.


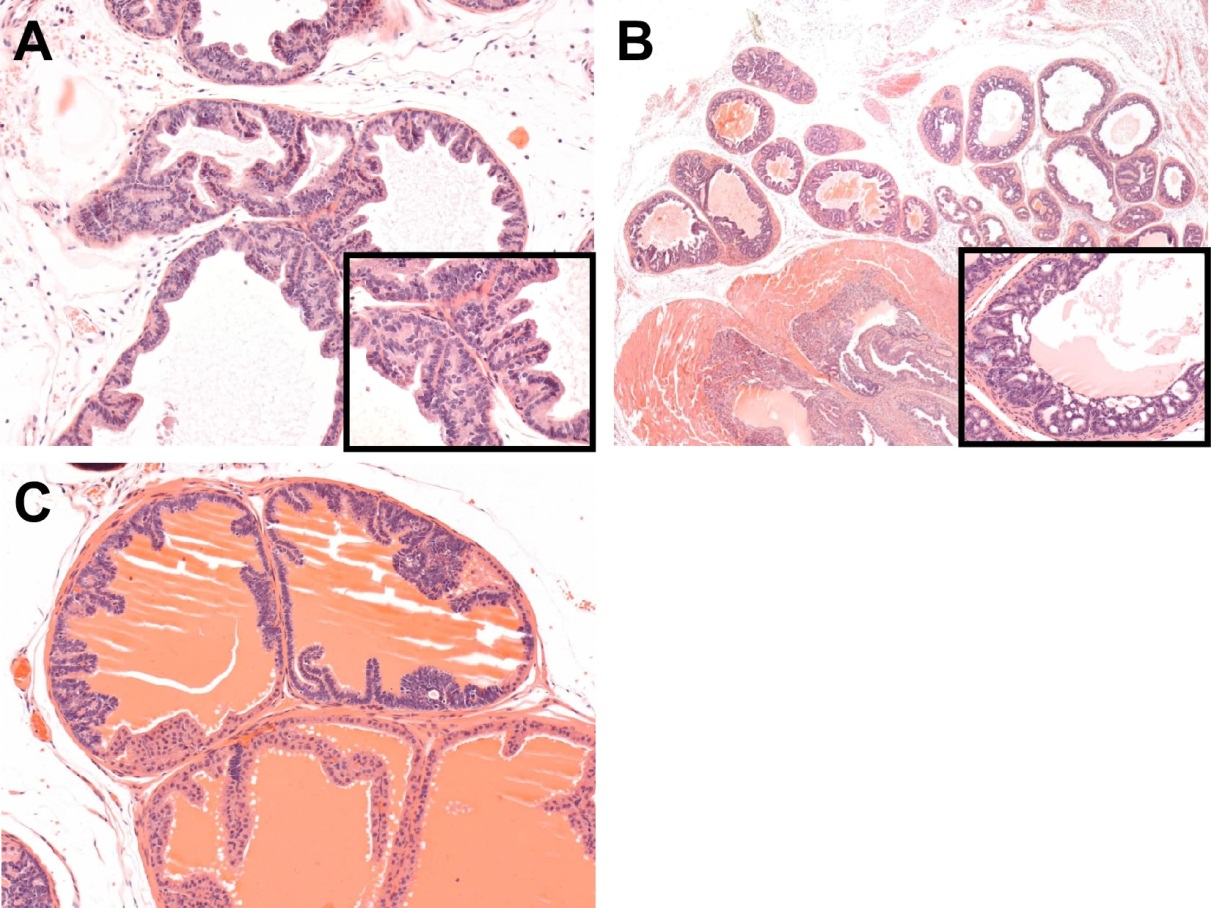


**Supplementary Figure 2**. Prostate histopathology in Gamitrinib-treated Group 1 TRAMP mice (24.9 weeks). Prostatic samples were analyzed by H&E staining and light microscopy. Representative cases of early (**A, C**) or advanced (**B**) PIN associated with modest inflammation are shown. **A**, ventral prostate; **B**, dorsal prostate; **C**, anterior prostate. Magnification, x40 (**A, B**); x200 (**C**). Insets, x400.


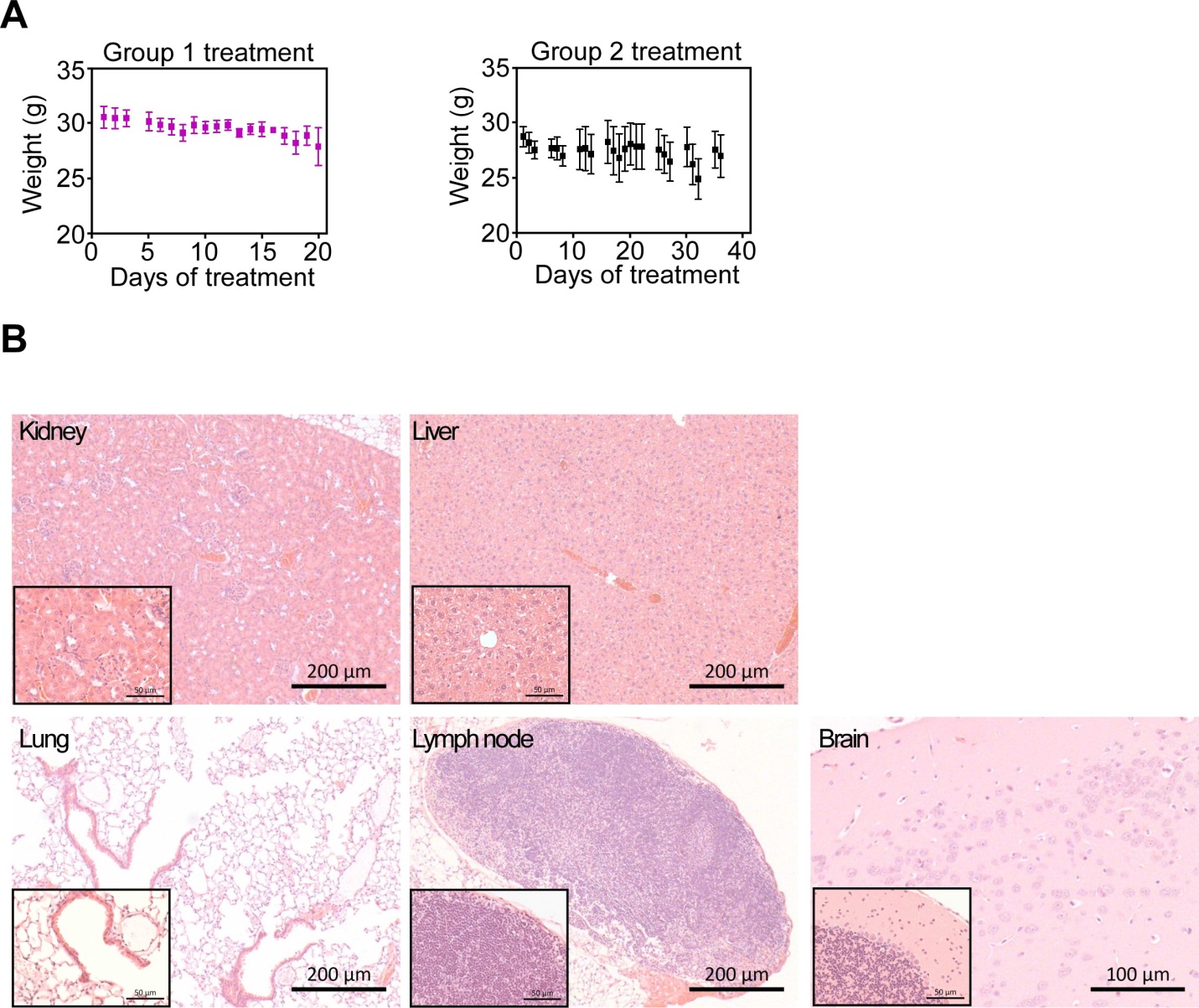


**Supplementary Figure 3**. Safety of Gamitrinib treatment of TRAMP mice. (**A**) TRAMP mice in Group 1 (*left*, 19.7 weeks of age) or Group 2 (*right*, 24.9 weeks of age) were weighed at the indicated time intervals during a 3- or 5-weeks course of systemic treatment with Gamitrinib, respectively. Data are the mean±SEM in the various groups with individual mice as units. (**B**) The indicated representative organs from Gamitrinib-treated TRAMP mice in Group 2 were collected at the end of the experiment, stained with H&E and analyzed histologically by light microscopy.
